# Natural Products with Pharmaceutical Activities

**DOI:** 10.3390/molecules30234557

**Published:** 2025-11-26

**Authors:** Soo Liang Ooi, Sok Cheon Pak

**Affiliations:** School of Dentistry and Medical Sciences, Charles Sturt University, Panorama Avenue, Bathurst, NSW 2795, Australia; sooi@csu.edu.au

## 1. Introduction

Natural products are bioactive molecules or compounds derived from natural sources that can be harnessed to develop therapeutic agents. Despite competition with synthetic molecules and biologics, drug discovery and development based on natural products remains a mainstay [1]. In recent years, advances in analytical chemistry, omics technologies, computational modelling, bioinformatics, automation, and artificial intelligence have accelerated the identification, characterisation, and pharmacological evaluation of bioactive natural compounds [2,3,4,5]. These developments have deepened our understanding of the mechanisms underlying the therapeutic effects of natural products and expanded their potential applications in modern medicine [6].

Despite these advancements, significant knowledge gaps and challenges remain—particularly in translating traditional ethnopharmacological knowledge and preclinical findings into clinically validated pharmaceutical agents [7]. Specifically, complex metabolites in plant- and marine-based natural products collectively contribute to their pharmacological properties, but hurdles still exist in the extraction and isolation of bioactive compounds for drug development [8]. Studying the pharmacokinetics of natural products to improve compound stability and bioavailability is also problematic due to the presence of multiple, diverse active compounds in their extracts [9]. In recent years, nanoparticle-based technology has shown tremendous potential in maximising drug delivery to ensure stability and bioavailability [10]. However, translational progress from the laboratory to viable production is often hindered by the complex interplay of regulatory and commercialisation hurdles [7].

The increasing demand for medicinal and aromatic plants from the pharmaceutical and cosmetic industries also intensifies their production, increasing the risk of overexploiting natural resources. Sustainable practices have become a necessity to ensure long-term economic, social, and environmental viability [11]. Sustainability in the natural product development life cycle includes harnessing bio-waste as a source of pharmacological compound discovery, developing sustainable methods for large-scale production, and eco-friendly and circular waste management [12,13,14,15]. However, with the predominant focus on the research and development of well-known compounds from medicinal and edible plants or food sources, many other promising natural product candidates have been left unexplored [16].

This Special Issue on “Natural Products with Pharmaceutical Activities” was conceived to address some of these knowledge gaps by showcasing current research and comprehensive reviews that explore natural compounds from diverse biological sources. The contributions highlight that, in addition to traditional medicinal plants, sustainable industrial byproducts and unconventional natural materials are promising resources for drug discovery. Novel approaches to the extraction, delivery, and production of natural pharmaceutical agents are also explored.

A total of thirteen papers, comprising eight original research articles and five reviews, were accepted after rigorous peer review. Collectively, these works demonstrate the growing integration of sustainability, innovation, and multidisciplinary approaches in natural product research.

## 2. An Overview of the Published Articles

### 2.1. Medicinal Plant Sources

Medicinal plants remain one of the most essential foundations for discovering new pharmaceutical agents; several papers in this Special Issue are dedicated to investigating these plants or their isolated bioactive compounds.

1′-Acetoxychavicol acetate (ACA) is a natural compound derived from the rhizomes of the Zingiberaceae family. Moriwaki et al. investigated the molecular mechanisms by which ACA inhibits the NF-κB signalling pathway in human lung adenocarcinoma A549 cells. The results showed that ACA lowered tumour necrosis factor (TNF)-α-induced NF-κB signalling pathway activity, which led to decreased intercellular adhesion molecule-1 (ICAM-1) expression by selectively downregulating the expression of the TNF receptor-associated factor protein (TRAF2) via the ubiquitin–proteasome system. In addition, ACA was also found to suppress the transcriptional process of NF-κB directly. The findings support ACA as a potential natural product candidate with anti-inflammatory and anticancer properties.

Naidoo and Khathi investigated the hepatoprotective properties of gossypetin, a natural flavonoid isolated from the calyx and flowers of Roselle (*Hibiscus sabdariffa*), in a pre-diabetic murine model. Gossypetin treatment significantly (*p* < 0.05) reduced liver triglycerides, liver weights, and plasma sterol regulatory binding element regulatory protein-1c, and improved liver antioxidant enzyme levels, compared to the pre-diabetic control. Decreased lipid droplet accumulation and improved tissue integrity were also observed in liver histology. The effects were found to be similar to those of metformin treatment. Notably, gossypetin was found to offer greater hepatoprotective effects in mice in the absence of dietary intervention.

*Salvia guevarae* is a species of flowering plant belonging to the Lamiaceae family, native to South America. Commonly cultivated for ornamental purposes, the species is also used in herbal remedies in folk medicine. Torres-Medicis et al. studied the antiproliferative and anti-inflammatory activities of nine neo-clerodane-type diterpenoids isolated from the dichloromethane extract of *S. guevarae* Bedolla & Zamudio leaves in vitro. Specifically, guevarain B and 6α-hydroxy patagonol acetonide showed moderate activity against the K562 human leukaemia cell line, whereas 2-oxo-patagonal, 6α-hydroxy patagonol acetonide, and 7α-acetoxy-ent-clerodan-3,13-dien-18,19:16,15-diolide inhibited nitric oxide production in RAW 264.7 macrophages.

Kanak et al. conducted a literature review on the application of Alchemilla species for skin health and dermatological disorders. Commonly known as lady’s mantle, Alchemilla is a genus of perennial herbaceous plants in the family Rosaceae. The review found that *Alchemilla* spp. exhibit antioxidant, anti-inflammatory, antimicrobial, and wound-healing activities. Extracts of Alchemilla plants were successfully used to treat dermatological issues including skin rashes, acne, stretch marks, eczema, psoriasis, and wrinkles. Nevertheless, more research is still needed to identify the most potent species or varieties, develop efficient extraction methods, and investigate efficacy in human application with appropriate formulations.

Sacha inchi (*Plukenetia volubilis*) is a plant native to South America that is renowned as a natural source of unsaturated fatty acids with enormous economic potential. Redjeki et al. reviewed the bioactivity, extraction methods, and encapsulation techniques of sacha inchi oil to inform nutraceutical and pharmaceutical development. The oil is rich in omega-3, omega-6, and omega-9, tocopherols, phytosterols, and polyphenols with bioactive activities including antioxidant, anti-inflammatory, antiproliferative, and neuroprotective effects beneficial to cardiovascular health. The stability of sacha inchi oil was a significant concern, with many current research studies discussing optimised extraction approaches and advanced microencapsulation techniques to enhance the efficacy of sacha inchi oil in application. Human research, especially clinical studies, remains lacking and is needed to validate the therapeutic benefits of the oil.

*Polyscias fruticosa*, commonly known as Ming aralia or “Vietnamese ginseng”, is used in traditional medicine in Southeast Asia for the treatment of a variety of conditions, such as fatigue, asthma, metabolic disorders, inflammation, and neurodegenerative diseases. A review by Śliwińska and Tomiczak offered a synthesis of the current understanding of the biotechnological methods, phytochemical diversity, and pharmacological activities of *P. fruticosa*. The review showed that *P. fruticosa* exhibits a complex phytochemical profile comprising triterpenoid saponins, flavonoids, phenolic acids, sterols, polyacetylenes, and fatty acid derivatives, with preclinical models demonstrating anti-inflammatory, antidiabetic, antiasthmatic, neuroprotective, and antiresorptive activities. Suspension cultures derived from callus tissues of *P. fruticosa* demonstrated biosynthetic competence for various classes of secondary metabolites, including triterpenoid saponins and phenolic compounds. Hence, the plant could be a promising alternative sustainable source of Araliaceae-type saponins for future pharmaceutical applications.

Pharmacokinetics software is increasingly being used in drug research to shorten development time and achieve cost savings. Silva et al. leveraged a range of computational tools to predict the pharmacological properties of withanolides, identified as a promising antineoplastic drug lead. Withanolides are naturally occurring C-28 ergostane steroidal lactones, commonly isolated from nightshade plants (Solanaceae family). This in silico study predicted their good oral absorption, low toxicity, and CYP3A4 metabolism with no inhibition of other P450 cytochromes; they were also unable to cross the blood–brain barrier. The molecules were predicted to show low inhibition of pharmacokinetic transporters and a high probability of nuclear receptor interactions, although they showed significant signs of aquatic ecotoxicity. Withanolides may therefore possess viability for oral drug development.

### 2.2. Sustainable Use of Bio-Wastes

Sustainability and circular use of resources are key emerging trends in natural product research.

Li et al. addressed environmental sustainability by exploring the potential use of *Cucumaria frondosa* tentacles, a waste product of the sea cucumber industry, as a natural antioxidant and anti-fatigue alternative. They found that the hydrolysates obtained through protease hydrolysis exhibited significant antioxidant capacity. The product was not toxic to mice and enhanced their exhaustive swimming time, significantly increased (*p* < 0.01) blood glucose and liver glycogen levels, reduced fatigue-related metabolites (*p* < 0.05), and elevated superoxide dismutase and glutathione peroxidase levels (*p* < 0.05). These findings support the potential utilisation of *Cucumaria frondosa* tentacles as a natural source of anti-fatigue products.

Xanthohumol is a prenylated chalcone found in hops (*Humulus lupulus*) and byproducts of beer production. Dlugosz et al. reviewed its mechanisms of action and therapeutic potential for the prevention of selected neurodegenerative diseases, including Alzheimer’s disease, Parkinson’s disease, and amyotrophic lateral sclerosis. Experimental evidence suggests that xanthohumol possesses potent antioxidant capabilities, beneficial for protecting neurons from oxidative damage, and could also alleviate neuroinflammation by inhibiting NF-κB, TNF-α, and IL-1β and reduce the chronic activation of microglia and astrocytes to prevent neuronal damage. Xanthohumol further influences the Nrf2/ARE neuroprotective pathway to enhance neuronal resilience, sustains neuronal energy metabolism by maintaining ATP production, and positively impacts synaptic plasticity, supporting memory enhancement; it could therefore have medical applications in the prevention and treatment of neurodegenerative diseases.

Roșcan et al. explored the potential of using plant waste, specifically from species such as red raspberry (*Rubus idaeus*) and cherry trees (*Prunus avium*, *Prunus serotina*, and *Prunus cerasus*), as a natural source of pharmaceutical agents. They found that plant waste from *R. idaeus*, *P. serotina*, *P. avium*, and *P. cerasus* contained complex bioactive compounds such as flavonoids, flavonols, tannins, cyanogenic glycosides, vitamins, aldehydes, and phenolic acids that exhibited pharmacological and pro-health properties, including antibacterial, antitumour, antidiabetic, anti-carcinogenic, neuroprotective, anti-inflammatory, antioxidant, and cardioprotective effects. A range of available extraction methods, from conventional ethanol extraction to more environmentally friendly solutions such as subcritical water extraction and supercritical CO_2_ extraction, were found in the literature. Also observed was an affinity for chromatographic methods for the isolation and purification of target compounds from the four plant sources, making them potentially valuable sources for numerous cosmetic, pharmaceutical, and food applications.

Xanthan gum is a biopolymer produced through the fermentation of sugars by the bacteria *Xanthomonas campestris*. Xanthan gum has extensive industrial applications and possesses many beneficial pharmaceutical properties. It is a non-toxic, biodegradable, and biocompatible substance that exhibits high viscosity and stability under a wide range of pH and temperature, and can thus be used as an excipient in many drug delivery systems, including sustained- or controlled-release formulations. Jonuskiene et al. investigated the use of renewable alternative carbon sources in the production of xanthan gum to replace sucrose and glucose and reduce environmental burden. The study demonstrated that L-glutamic acid was a suitable source for 72 h bacterial fermentation, yielding the highest production. However, other renewable natural resources, including avocado hydrolysates and lemon peels, could also achieve high xanthan gum yields with only 24 h of fermentation and exhibited the highest antioxidant activity. Thus, these renewable resources could be used to improve the sustainability of xanthan gum production.

### 2.3. Diverse Natural Sources

The remaining two papers broaden the scope of natural product research beyond traditional plant-based systems.

To manage foodborne illnesses caused by pathogens such as *Salmonella enterica* and *Escherichia coli*, Maione et al. assessed the viability of phenyllactic acid produced by lactic acid bacteria as an anti-microbial agent in vitro, which was found to be non-toxic to intestinal cells and effective in inhibiting biofilm formation and bacterial planktonic growth, with minimum inhibitory concentrations ranging from 2 to 2.75 mg/mL. Experimenting with real food, the application of phenyllactic acid to minced beef significantly (*p* < 0.05) lowered microbial populations compared to controls. It was also found that phenyllactic acid has the potential to prevent intestinal bacterial colonisation, making it a promising agent not only for controlling microbial contamination in food processing but also for making the intestinal microenvironment less hospitable for harmful bacteria.

Amber is a type of organic gemstone formed from the fossilisation of ancient tree resin. Yodweerapong et al. examined the T cell inhibition activities of kujigamberol, a dinorlabdane compound isolated from amber mined from the Kuji region of Japan. This compound was found to lower IFN-γ production and IL-2 mRNA expression in murine lymphoma BW5147 T-cell and cytotoxic T-cell line CTLL-2. Kujigamberol also reduced nuclear factor of activated T cell (NFAT)-dependent transcription in human embryonic kidney 293 T cells and suppressed T-cell activities through interfering with the binding of NFATc2 to the IFN-γ and IL-2 promoters. Hence, it could be a potential lead for immunosuppressive agents.

## 3. Conclusions

The studies published in this Special Issue collectively showcase the remarkable diversity of natural sources for pharmaceutical discovery, ranging from traditional medicinal plants and sustainable industrial byproducts to rare materials like amber.

They reflect the ongoing global commitment to unlocking nature’s pharmacological potential while embracing sustainability, biotechnological innovation, and interdisciplinary collaboration. Together, these contributions reinforce that natural product research remains a vibrant, evolving field that continues to offer promising leads for the development of novel therapeutics addressing modern health challenges.
